# Accuracy and learning curve of imageless robotic-assisted total knee arthroplasty

**DOI:** 10.1016/j.jor.2024.12.029

**Published:** 2025-01-02

**Authors:** Francesco Bosco, Giuseppe Rovere, Carmelo Burgio, Giorgia Lo Bue, Claudio Domenico Cobisi, Riccardo Giai Via, Ludovico Lucenti, Lawrence Camarda

**Affiliations:** a Department of Precision Medicine in Medical, Surgical and Critical Care (Me.Pre.C.C.), University of Palermo, 90133, Palermo, Italy; b Department of Orthopaedics and Traumatology, G.F. Ingrassia Hospital Unit, ASP 6, 90131, Palermo, Italy; c Department of Orthopaedics and Traumatology, Fondazione Policlinico Universitario A. Gemelli IRCCS, Università Cattolica del Sacro Cuore, 00168, Rome, Italy; d Department of Clinical Science and Translational Medicine, Section of Orthopaedics and Traumatology, University of Rome "Tor Vergata", 00133, Rome, Italy; e Adult Reconstruction and Joint Replacement Service, Hospital for Special Surgery, 10021, New York, NY, USA; f Department of Orthopaedic Surgery, Centro Traumatologico Ortopedico (CTO), University of Turin, Via Gianfranco Zuretti, 29, 10126, Turin, Italy

**Keywords:** Robotic, Total knee arthroplasty, RA-TKA, TKA, Knee, Osteoarthritis

## Abstract

**Background:**

Total knee arthroplasty (TKA) is widely used to manage severe knee osteoarthritis. However, conventional TKA (C-TKA) often leaves patients dissatisfied due to suboptimal alignment and soft-tissue balance. Robotic-assisted TKA (RA-TKA), particularly with imageless systems like the NAVIO Surgical System, promises enhanced accuracy and improved outcomes. This study aims to validate the accuracy of RA-TKA in achieving functional alignment (FA) and to explore the learning curve associated with this technique.

**Materials and methods:**

A retrospective analysis included 101 patients undergoing RA-TKA with the NAVIO system from July 2021 to April 2024. Data on alignment angles, gap balance, and surgical times were analyzed. Patients were categorized by preoperative coronal alignment (valgus, neutral, and varus), with subgroups within the varus category. Accuracy was defined as deviations ≤3° for alignment and ≤1 mm for gap balance. Learning curve trends were analyzed using segmented regression.

**Results:**

The study demonstrated a mean alignment error of 1.18° (±1.21) and a gap balance accuracy of 84 %, with no significant differences across knee morphologies. The RA-TKA system achieved an overall implant alignment accuracy rate of 95 %. Varus knees with greater deformities (>6°) showed comparable or superior accuracy to less severe cases. Surgical time averaged 72.3 min (±5.6), with significant reductions observed after the first 11 cases, reflecting procedural efficiency improvements without compromising accuracy.

**Conclusion:**

The RA-TKA reliably achieves precise FA across diverse knee morphologies with a rapid learning curve. Future studies should evaluate long-term outcomes and implant survivorship to confirm these promising findings.

**Level of evidence:**

IV.

## 1 Introduction

Total knee arthroplasty (TKA) is the most common surgical procedure for managing severe knee osteoarthritis, offering significant pain relief, improved joint function, and enhanced quality of life for patients. However, approximately one-fifth of patients continue to report dissatisfaction following conventional total knee arthroplasty (C-TKA).^1^, ^2^, ^3^, ^4^ The success of TKA depends on various intraoperative factors, including limb alignment, implant sizing, soft-tissue balance, and the accuracy of implant positioning.

Robotic-assisted total knee arthroplasty (RA-TKA) has been introduced to address these challenges and improve clinical outcomes, aiming to enhance precision and patient satisfaction.^5^, ^6^, ^7^, ^8^ Early follow-up studies suggest that RA-TKA offers improved short-term outcomes compared to C-TKA, with fewer radiographic outliers and a lower risk of iatrogenic soft tissue injuries.^7^, ^8^, ^9^

RA-TKA employs advanced computer software to create a personalized, three-dimensional model of the patient's knee joint. Robotic systems can be divided into image-based and imageless systems, which differ primarily in data acquisition methods. Image-based systems use preoperative imaging, such as computed tomography (CT) scans, to obtain detailed anatomical data, enabling personalized planning and implant selection. While this approach can theoretically enhance accuracy,^8^^,^^10^ it also raises concerns about increased radiation exposure and higher procedural costs.^11^, ^12^, ^13^ Conversely, imageless systems rely on intraoperative mapping with a handheld probe to generate a virtual 3D model of the knee, but they may be considered less accurate by some.^9^^,^^11^^,^^14^, ^15^, ^16^, ^17^, ^18^

A novel alignment strategy, known as functional alignment (FA), has recently emerged as an alternative to the traditional mechanical alignment (MA). Unlike MA, which prioritizes the mechanical axis of the knee, FA focuses on optimizing the interaction between prosthetic components and surrounding soft tissues during dynamic movement. By integrating the principles of measured resection and gap balancing, FA aims to restore the original biomechanics of the limb while respecting the patient's natural soft tissue envelope.^19^, ^20^, ^21^ Accurate functional alignment enhances strength, coordination, and mobility, improving postoperative outcomes and patient satisfaction.^20^, ^21^, ^22^ Conversely, misalignment can result in compensatory movement patterns, altered gait mechanics, and increased strain on adjacent joints and soft tissues.^23^, ^24^, ^25^, ^26^

This study aims to validate the accuracy of a robotic system in achieving planned functional alignment in TKA by assessing the differences between the preoperative plans and postoperative outcomes in terms of alignment angles and gap balance. Furthermore, it seeks to explore how variations in knee morphology influence the system's precision and to evaluate the learning curve of RA-TKA by analyzing the relationship between surgical experience, procedural accuracy, and operative time. We hypothesize that RA-TKA delivers superior alignment accuracy and gap balance, with certain native knee alignments contributing to greater precision, and that increased surgical experience significantly enhances both procedural efficiency and clinical outcomes.

## 2 Materials and methods

### 2.1 Study design

This retrospective study evaluated a consecutive series of patients diagnosed with end-stage knee osteoarthritis who underwent primary TKA using robotic-assisted surgery. The procedures were performed at the Orthopedic and Traumatology Unit of AOUP ‘P. Giaccone’, Palermo, Italy, between July 2021 and April 2024.

### 2.2 Inclusion and exclusion criteria

Inclusion criteria for the study included patients with a diagnosis of end-stage knee osteoarthritis based on the Ahlbäck Classification,^27^ older than 18 years, and underwent knee replacement surgery using the NAVIO Surgical System (Smith & Nephew, Memphis, USA). Exclusion criteria included failure to record intraoperative surgical parameters, diagnosis of inflammatory arthropathies, functional incompetence of the ligaments, and severe bone defects such as valgus and varus deformities exceeding 20°.

### 2.3 Surgical technique

All TKAs were performed by a senior surgeon (L.C.) using the NAVIO Surgical System (Smith & Nephew, Memphis, USA). The procedures adhered to FA principles and utilized the JOURNEY™ II BCS Knee System implant design. A standard mid-vastus approach was employed for all procedures. After joint exposure, tracking arrays were mounted on the distal femur and proximal tibia by inserting pins to establish stable reference points for navigation. The patient-specific anatomical data were registered using the robotic system by collecting key points, including the malleoli for tibial alignment, the knee center, and the femoral head center, to reconstruct a precise 3D limb model. The knee morphology was mapped by probing the condylar surfaces, ensuring accurate anatomical landmarking. Kinematic analysis was then performed by passively flexing and extending the knee to register its natural motion, capturing dynamic data such as the rotational axis and the flexion-extension ROM. During this process, valgus and varus stress tests were applied throughout the full range of extension and flexion to tension the medial and lateral structures, allowing an assessment of the soft tissue balance.

**Fig. 1:**
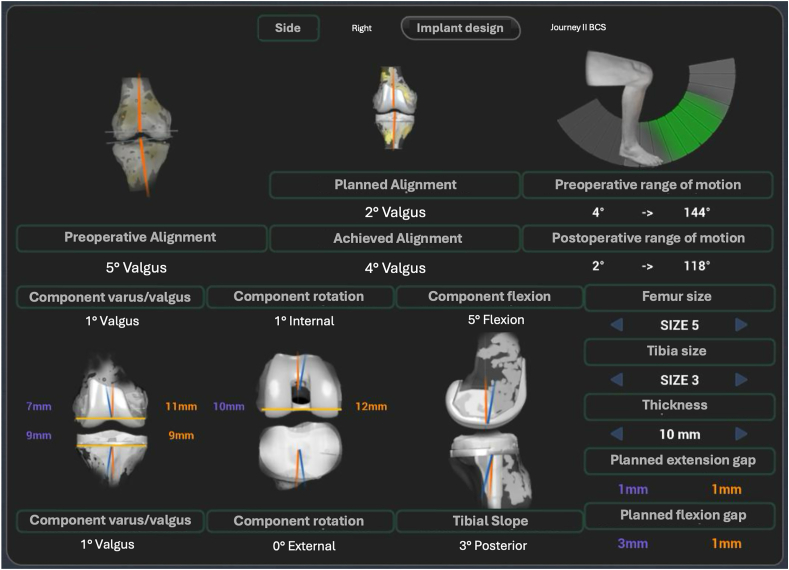
Graphical representation of intraoperative data collected during a total knee arthroplasty (TKA) performed with the NAVIO robotic surgical system (Smith & Nephew). The image illustrates preoperative, planned, and achieved alignment values, along with an analysis of the range of motion (ROM) and joint gap balancing. The data also detail the rotation and positioning of the femoral and tibial components, with visual feedback on the adaptation of the JOURNEY™ II BCS Knee System implant, performed according to functional alignment (FA) principles. The system enabled real-time personalized optimization through visual feedback and robotic assistance, ensuring precision in bone preparation and soft tissue balancing for an optimal functional outcome.

The robotic software provided a 3D virtual representation of the patient's anatomy, enabling the surgeon to customize implant positioning in real time to achieve FA. The system's graphical interface displayed continuous feedback on alignment and gap balance, allowing iterative adjustments to virtual implant placement and bone resection plans without requiring extensive ligament releases. Bone preparation was performed using the NAVIO handpiece, a robotic-assisted device that ensured precise resections of the distal femur and proximal tibia as per the preoperative plan. Trial implants were then positioned to confirm alignment and joint balance, with minor resections or soft tissue adjustments made as necessary to achieve optimal results. Once the surgeon confirmed proper alignment and balance, cemented implants were applied to the prepared bone surfaces, and the final implant position and gap balance were verified throughout the entire ROM to ensure the best possible functional outcome. At the end of the procedure, the system enabled visualization of the differences in alignment between the preoperative status, the planned configuration, and the achieved outcome, providing valuable insights for postoperative evaluation (Fig. 1).

### 2.4 Data collection

**Fig. 2:**
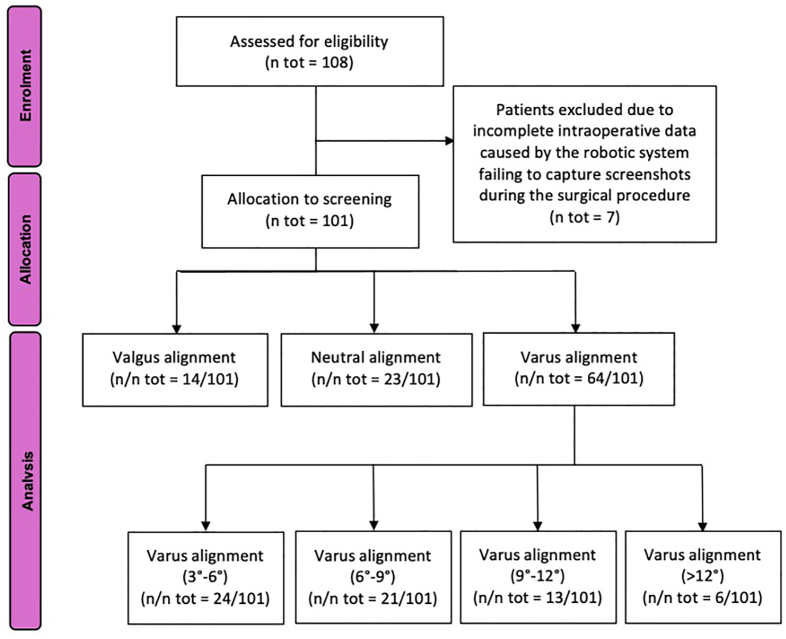
Flowchart illustrating patient enrollment, allocation, and analysis. N = number of evaluation cases: ° = degree.

The study enrolled a total of 108 patients who underwent primary RA-TKA using the NAVIO Surgical System (Smith & Nephew, Memphis, USA). Of these, seven patients were excluded due to incomplete intraoperative data caused by the robotic system failing to capture screenshots during the surgical procedure. As a result, these cases were deemed unusable for the study, leaving 101 patients with complete intraoperative data eligible for analysis (Fig. 2).

Data were recorded by the robotic surgical system at each procedural step. This included preoperative, planned, and achieved alignment values and gap balance measurements. In addition, the system documented preoperative and postoperative range of motion, bone resections, and details about the specific implants used. The positioning of the femoral and tibial implants was evaluated in terms of angles, rotation, and flexion, with particular attention to femoral flexion and tibial posterior slope.

Clinical data were retrospectively collected, including patients' demographic in-formation, surgical records, and pre-and postoperative knee X-rays.

### 2.5 Data analysis

Patients were categorized preoperatively based on their coronal alignment into three groups: valgus (HKA < −3°), neutral (−3° ≤ HKA ≤3°), and varus (HKA >3°). Further sub-classifications were made to assess the system's accuracy in this subgroup within the varus group. Patients with a native knee alignment were divided into the following categories: light varus (3°–6°), mild varus (6°–9°), severe varus (9°–12°), and very severe varus (>12°). Accuracy was evaluated by calculating the difference between planned and achieved values for both alignment and gap measurements. Accuracy thresholds were set at 3° for alignment and 1 mm for gap balance. Additionally, surgical time was specifically recorded to measure the duration required for robotic system utilization.

### 2.6 Ethical approval

This retrospective study, which investigated an established surgical procedure, fully complied with the ethical principles outlined in the 1964 Declaration of Helsinki and its subsequent amendments. All participants were thoroughly informed about the study's nature and objectives and provided written informed consent prior to undergoing the surgical procedure. The study was conducted under the approved research protocol titled “Robotic Knee Prosthesis” and received formal approval from the Local Ethics Committee Palermo 1 (Minutes No. 03/2024).

### 2.7 Statistical analysis

Statistical analysis was conducted using R software (version 4.1.3, R Core Team, Vienna, Austria). Descriptive statistics, including mean, standard deviation, and range, were applied to summarize the measurement values for component placement. Differences between planned and achieved alignment and gap balance values were calculated and analyzed to assess the accuracy of the robotic system. Paired and independent sample t-tests were performed to compare mean differences between groups, such as planned versus achieved values and subgroups with distinct knee morphologies. A p-value of less than 0.05 was considered statistically significant. The learning curve associated with RA-TKA was evaluated, focusing on accuracy and surgical times. To assess the stabilization of surgical times and identify the learning curve, a segmented regression analysis was performed. This statistical method divides the dataset into two linear segments separated by a change point, which was determined by minimizing the Residual Sum of Squares (RSS) across multiple potential breakpoints. This approach identified the transition from the learning phase to the stabilization phase, highlighting trends in surgical performance and procedural efficiency over time, and providing valuable insights into the progression of expertise with the robotic system.

## 3 Results

### 3.1 Demographic characteristics

The study included a total of 101 patients, with demographic data summarized in Table 1. The mean age of the cohort was 70.9 years (±7.0), with a higher proportion of females (61 cases) compared to males (40 cases). The mean BMI was 29.7 kg/m^2^ (±3.7). Regarding the side of the affected limb, the right side was more commonly involved (65 cases) compared to the left (36 cases).

**Table 1:** Demographics characteristics of the included patients.

Parameters	Mean	SD
Age (years)	70.9	7.0
Sex (M − F)	40–61∗	
BMI (kg/m^2^)	29.7	3.7
Side (Right - Left)	65–36∗	

SD = standard deviation; M = male; F = female; BMI = Body mass index; kg = kilogram; m = meter; ∗ = number of evaluation cases.

### 3.2 Alignment and gap balance accuracy

The study demonstrated that FA was achieved with a mean alignment error of 1.18° (±1.21), resulting in an overall implant placement accuracy rate of 95 %. Similarly, the planned gap balance was achieved with 84 % accuracy, and the average error in gap measurement was 0.37 mm (±0.46). Detailed results on alignment and gap balance errors, bone resection measurements, and implant positions are summarized in Table 2.

**Table 2:** Summary of alignment accuracy, gap balance, and implant placement results.

Parameters		Total patients		Valgus		Neutral		Varus	
		Mean	SD	Mean	SD	Mean	SD	Mean	SD
Alignment	Preoperative^a^	4.3	6.4	−7.1	2.7	0.3	2.0	8.2	3.4
	Planned^a^	1.3	2.6	−2.9	1.5	−0.1	1.7	2.8	1.6
	Achieved^a^	1.4	3.2	−3.9	2.3	0.3	2.2	2.9	2.1
Gap accuracy evaluation	Medial gap in extension			1.4	0.5	1.2	0.5	1.0	0.5
	Lateral gap in extension			1.1	0.5	1.3	0.6	2.0	1.0
	Medial gap in flexion			1.2	0.7	1.2	0.8	1.1	0.7
	Lateral gap in flexion			1.6	1.1	2.0	1.1	2.2	1.0
Coronal plane angle	Femoral component^a^	0.4	1.7	−2.1	1.1	−0.3	0.9	1.2	1.3
	Tibial component^a^	1.0	1.3	−0.6	1.2	0.6	1.0	1.5	1.0
ROM	Preoperative ROM in extension	0.9	6.2	1.2	5.7	−1.8	4.4	1.7	6.6
	Preoperative ROM in flexion	132.9	8.3	132.6	9.3	134.2	9.7	132.4	7.5
	Postoperative ROM in extension	1.8	1.5	2.0	2.0	1.4	1.4	1.9	1.5
	Postoperative ROM in flexion	118.6	1.6	118.4	0.9	118.7	2.5	118.6	1.2
Components position	Femoral component rotation^b^	2.3	2.0	0.5	2.5	2.7	1.0	2.6	1.9
	Tibial component rotation^b^	0.1	1.7	0	0	0.7	2.2	−0.2	1.7
	Femoral component flexion	3.7	1.4	4.3	1.7	3.7	1.3	3.6	1.4
	Tibial component slope	2.9	0.5	2.8	0.8	2.8	0.7	3.0	0.4
Femoral and tibial cuts	Medial thickness of femoral cut (mm)	9.1	1.8	10.0	1.0	9.2	1.6	8.9	2.0
	Lateral thickness of femoral cut (mm)	7.9	1.5	6.6	1.7	7.5	1.3	8.4	1.3
	Medial thickness of tibial cut (mm)	6.5	2.2	8.0	1.2	7.3	1.6	5.9	2.3
	Lateral thickness of tibial cut (mm)	9.4	1.6	7.4	1.8	9.2	1.5	9.9	1.3
	Medial thickness of posterior cut (mm)	11.5	1.7	11.6	1.4	10.9	1.4	11.7	1.8
	Lateral thickness of posterior cut (mm)	9.2	1.7	10.0	2.2	9.0	1.5	9.1	1.6

ROM = range of motion; mm = millimeter; SD = standard deviation.

a = Varus values are expressed as positive numbers, while valgus values are expressed as negative numbers.

b = External rotation is expressed as a positive number.

### 3.3 Alignment accuracy by knee morphology

Among the different preoperative alignment groups, the mean alignment error was 1.0° (±1.25) for the valgus group, 1.0° (±1.18) for the neutral group, and 1.28° (±1.19) for the varus group, with no statistically significant differences in alignment accuracy between these knee morphologies. Deviation from the planned gap balance remained below 0.5 mm in both valgus and varus knees, irrespective of the severity of the native alignment. In the neutral knees, a slightly reduced accuracy was observed in flexion, with a median error of 0.66 mm (±0.76) medially and 0.7 mm (±0.84) laterally; however, these deviations did not show statistical significance compared to the control. Further details on accuracy values by knee phenotype are provided in Table 3.

**Table 3:** Comparison of knee morphotypes: Varus, valgus, and neutral.

Knee morphotypes	Varus (n = 64)		Valgus (n = 14)		T-test	p.value
	Mean	SD	Mean	SD		
Alignment	1.28	1.19	1	1.25	0.79	0.43
Medial gap in extension	0.29	0.19	0.34	0.14	1.03	0.31
Lateral gap in extension	0.32	0.35	0.28	0.33	0.43	0.66
Medial gap in flexion	0.31	0.38	0.26	0.28	0.51	0.61
Lateral gap in flexion	0.31	0.34	0.33	0.39	0.24	0.81

Knee morphotypes	Valgus (n = 14)		Neutral (n = 23)		T-test	p.value
	Mean	SD	Mean	SD		
Alignment	1	1.25	1	1.18	0	1
Medial gap in extension	0.34	0.14	0.43	0.56	0.54	0.59
Lateral gap in extension	0.28	0.33	0.39	0.34	0.98	0.33
Medial gap in flexion	0.26	0.28	0.66	0.76	1.88	0.07
Lateral gap in flexion	0.33	0.39	0.70	0.84	1.54	0.13

Knee morphotypes	Varus (n = 64)		Neutral (n = 23)		T-test	p.value
	Mean	SD	Mean	SD		
Alignment	1.28	1.19	1	1.18	0.97	0.33
Medial gap in extension	0.29	0.19	0.43	0.56	1.74	0.08
Lateral gap in extension	0.32	0.35	0.39	0.34	0.80	0.43
Medial gap in flexion	0.31	0.38	0.66	0.76	2.79	0.01
Lateral gap in flexion	0.31	0.34	0.70	0.84	3.12	0.01

N = number of evaluation cases; SD = standard deviation.

### 3.4 Varus subgroup analysis

Analysis of the varus subgroup revealed a mean alignment error of 1.45° (±1.18) for varus knees with angles exceeding 6°. Although accuracy appeared slightly reduced, this difference was not statistically significant compared to the control group (p = 0.068). A more analysis found that patients with mild varus morphology (3°–6°) had an average error of 1.52° (±1.37), while those with severe (9°–12°) and very severe varus (>12°) showed better accuracy, with average errors of 1.39° (±1.39) and 1.33° (±1.03), respectively. These findings suggest that the robotic system may perform more accurately with greater degrees of varus, although the differences among subgroups were not statistically significant (p > 0.05). Detailed results of the varus subgroup analysis are reported in Table 4.

**Table 4:** Detailed comparison of knee morphotypes in different varus ranges.

Knee morphotypes	Varus (3°–6°) (n = 24)		Varus (6°–9°) (n = 21)		Varus (9°–12°) (n = 13)		Varus (>12°) (n = 6)	
	Mean	SD	Mean	SD	Mean	SD	Mean	SD
Alignment	1	1.22	1.52	1.36	1.38	0.96	1.33	1.03
Medial gap in extension	0.27	0.21	0.29	0.23	0.3	0.12	0.33	0.15
Lateral gap in extension	0.29	0.19	0.45	0.55	0.21	0.18	0.25	0.16
Medial gap in flexion	0.34	0.46	0.23	0.19	0.38	0.53	0.32	0.13
Lateral gap in flexion	0.31	0.47	0.32	0.32	0.3	0.18	0.32	0.15

N = number of evaluation cases; ° = degree; SD = standard deviation.

### 3.5 Surgical time and learning curve

**Fig. 3:**
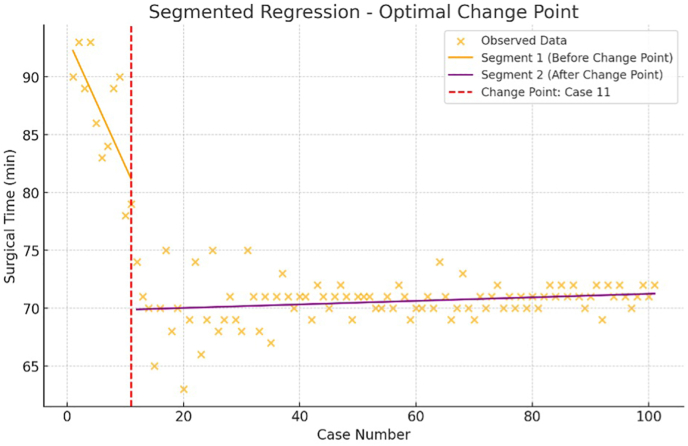
Segmented regression analysis assessing the learning curve of surgical time for robot-ic-assisted total knee arthroplasty (RA-TKA).

The mean surgical time for the procedures was 72 min (±18.4). A segmented regression analysis of the learning curve indicated that while accuracy showed no statistically significant improvement over time, surgical time significantly decreased after the first 11 cases. The surgical time learning curve is illustrated in Fig. 3.

## 4 Discussion

The most important finding of this study was the demonstration of the high accuracy of the NAVIO robotic system in achieving FA, with an implant alignment accuracy of 95 % and a gap balance accuracy of 90 %. These results provide strong evidence supporting the reliability of this imageless system in precisely achieving planned alignment without requiring preoperative CT scans. Notably, no significant differences in alignment accuracy were observed across the three native knee phenotypes, indicating the system's adaptability to diverse knee morphologies. While gap balance accuracy was slightly lower in neutral knees, valgus and varus knees consistently achieved accurate gap balance in both extension and flexion. These findings further contribute to the growing body of evidence supporting the efficacy of robotic systems in managing various knee alignments, underscoring their value in modern TKA procedures.

The traditional goal of achieving a 0° hip-knee-ankle (HKA) axis in coronal alignment, known as MA, has recently been supplemented by the introduction of new alignment strategies.^19^^,^^20^ A standardized MA approach, while widely used, raises concerns as it may fail to align with the natural anatomy and kinematics of each patient's knee, potentially influencing postoperative satisfaction and functional outcomes.^21^^,^^28^ FA is a recently developed concept that positions and orients the knee prosthesis to restore the natural biomechanics and alignment of the patient's knee. This method prioritizes how the knee interacts with surrounding soft tissues during movement, aiming to ensure optimal functionality within the context of overall body mechanics.^24^^,^^26^

Notably, Liu et al.^28^ reported that functionally aligned TKA resulted in superior 1-year postoperative patient-reported outcomes compared with mechanically aligned TKA, emphasizing its ability to enhance the precision of RA-TKA. While many studies have demonstrated the NAVIO surgical system's reliability in achieving neutral coronal alignment,^14^, ^15^, ^16^, ^17^ our study focused on its ability to achieve FA across diverse knee morphologies.

FA has also been investigated in other robotic systems. For instance, Zambianchi et al.^22^ demonstrated the effectiveness of an image-based RA-TKA in restoring the native joint line through a tibia-based FA approach, achieving consistent alignment accuracy across different knee phenotypes. Similarly, Bollars et al.^18^ highlighted the capability of an imageless, semi-autonomous robotic system to achieve FA with precision, regardless of the native alignment, by allowing intraoperative adjustments based on real-time an-atomical mapping and soft tissue balancing. These findings, in combination with our results, underscore the utility of robotic-assisted systems in delivering personalized alignment strategies for TKA.

Although there is ongoing debate regarding the optimal limb alignment strategy in TKA, it remains clear that achieving a well-balanced TKA is essential for both long-term implant survival and patient satisfaction.^23^, ^24^, ^25^ Accurate FA ensures that prosthetic components interact appropriately with the surrounding soft tissues, promoting optimal ligament tension and muscle function. This alignment is crucial to mitigate compensatory movements that may lead to overactivation of some muscle groups and underutilization of others, ultimately causing weakness and joint instability.^24^^,^^25^ Additionally, tibial component malalignment is a well-documented cause of early TKA failure.^29^

C-TKA, even when performed with precision, has been associated with a significant risk of outliers, ranging between 17.4 % and 25 %.^2^^,^^7^^,^^8^ Robotic-assisted systems were developed to address these limitations by providing enhanced control over component positioning. These systems allow surgeons to perform highly accurate bone resections and implant placements guided by real-time feedback, reducing alignment errors by up to 30 %.^8^^,^^9^^,^^12^

A key distinction among RA-TKA systems lies in their preoperative data input. Image-based robotic systems require preoperative CT scans, whereas imageless systems rely solely on intraoperative data, eliminating the need for preoperative imaging. While preoperative imaging has been associated with longer procedural times, higher radiation exposure, and increased costs,^12^^,^^13^ its impact on surgical accuracy remains a topic of debate. Some studies suggest that preoperative imaging enhances precision^10^^,^^12^; however, others argue that intraoperative mapping alone achieves comparable accuracy without the added complexities and costs.^11^^,^^13^ For example, Yee et al.^11^ concluded that both image-based and imageless robotic systems demonstrated comparable accuracy in coronal alignment, with imageless systems showing superior accuracy in sagittal tibial alignment. Similarly, Mayne et al.^30^ reported that the ROSA robotic system achieved similar component positioning accuracy regardless of the input method, highlighting the potential of imageless systems to reduce radiation exposure without compromising out-comes.

Our study supports these findings by demonstrating that imageless handheld robotic systems effectively reproduce planned limb alignment and gap balance, achieving ac-curacy levels comparable to those reported for image-based systems.^8^^,^^10^^,^^12^ This im-ageless approach simplifies the surgical workflow and eliminates the need for preoperative imaging, reducing procedural complexity and minimizing radiation exposure. Alongside other findings,^14^, ^15^, ^16^, ^17^, ^18^ our study adds to the growing evidence of the excellent accuracy achieved by robotic-assisted systems. For instance, Rossi et al.^14^ reported deviations of less than 1 mm and 1° in achieving planned alignment using the imageless ROSA system. Adamska et al.^15^ showed that NAVIO and CORI systems achieved alignment errors within 1.5° of scheduled values, and Savov et al.^16^ demonstrated that NAVIO achieved planned HKA alignment within 2°.

Importantly, our results indicated no learning curve effect in terms of accuracy, consistent with the broader literature. Collins et al.^31^ reported that robotic-assisted systems produced accurate coronal alignment in over 93 % of patients without a learning curve effect for accuracy. These findings highlight the reliability of robotic-assisted systems, irrespective of surgeon experience, in maintaining alignment precision.

Studies have consistently reported prolonged surgical durations for RA-TKA com-pared to C-TKA, particularly during the early adoption phase.^7^^,^^9^^,^^12^^,^^32^ In our study, the average robotic surgical time was 72.3 min (±5.6), with a learning curve analysis showing a significant reduction in surgical time after 11 cases. This aligns with findings by Collins et al.,^31^ who observed that robotic-assisted procedures initially took 30–40 min longer than C-TKA. Similarly, Savov et al.^16^ reported a learning curve plateau after 11 cases, reinforcing the notion that surgical efficiency improves with experience. Notably, advancements in robotic technology are promising; for example, the second-generation CORI system has demonstrated significantly shorter surgical times, averaging 55.0 ± 30.2 min, compared to 67.3 ± 24.3 min with the NAVIO system,^33^ highlighting the potential for further procedural optimization in the future.

This study has several strengths. The relatively large cohort size enhances its level of evidence compared with similar case series. Despite its retrospective design, all clinical data were collected through the internal robotic system registry at the time of surgery, ensuring data accuracy and completeness. Furthermore, all procedures were performed by the same surgeon using a consistent surgical technique and implant, reducing the influence of intraoperative variability.

This study has several limitations that should be acknowledged. First, its retrospective design inherently carries limitations, including potential biases and the inability to establish causality. Second, the results and measurements were derived directly from the NAVIO Surgical System, necessitating further independent research to validate the high level of accuracy reported. Third, the absence of a control group restricts the ability to fully evaluate the impact of independent variables, such as patient-specific factors or alternative surgical techniques. Another limitation of this study is the inability to perform subgroup analyses for patients with valgus knee morphotypes. The small sample size in this category did not allow for a statistically adequate evaluate. Finally, the study groups were slightly inhomogeneous, with variations in the patient's native knee morphotypes, which may have influenced the analysis. Addressing these limitations in future research with controlled, prospective designs and more homogenous populations will provide more robust evidence.

## 5 Conclusions

This study confirms that the imageless robotic system accurately achieves planned functional alignment with no learning curve effect on implant placement accuracy. The system ensures precise positioning while minimizing radiation exposure by utilizing intraoperative mapping and real-time soft tissue balancing. Additionally, its effectiveness across various knee phenotypes underscores its versatility. Future research should focus on evaluating long-term implant survivorship, patient satisfaction, and alignment-related failures to further validate its clinical benefits.

## CRediT authorship contribution statement

**Francesco Bosco:** Conceptualization, Writing – original draft. **Giuseppe Rovere:** Investigation, Visualization. **Carmelo Burgio:** Writing – original draft, Data curation, Methodology, Investigation. **Giorgia Lo Bue:** Data curation, Methodology. **Claudio Domenico Cobisi:** Visualization, Supervision. **Riccardo Giai Via:** Visualization. **Ludovico Lucenti:** Visualization, Supervision. **Lawrence Camarda:** Supervision.

## Guardian/patient's consent

Not Applicable.

## Institutional review board statement

Research protocol titled “Robotic Knee Prosthesis” has been formally approved by the Local Ethics Committee Palermo 1 (Minutes No. 03/2024).

## Ethical statement

The study was conducted following the ethical standards of the Declaration of Helsinki (1964).

Research protocol titled “Robotic Knee Prosthesis” has been formally approved by the Local Ethics Committee Palermo 1 (Minutes No. 03/2024).

## Funding statement

This research did not involve any specific grants from commercial, public, or non-profit sector funding agencies.

## Conflict of interest statement

On behalf of all authors, the corresponding author states that there is no conflict of interest.
